# Behavioral and electrophysiological evaluation of the efficacy of* Origanum vulgare *essential oil as anesthesia in Amazonian zootechnical fish

**DOI:** 10.1007/s10695-026-01674-4

**Published:** 2026-03-28

**Authors:** Axell Lins, Rômulo Augusto Feio Farias, Júlia Schneider Santiago, Luiz Fernando Duarte de Andrade Júnior, Antonio José Souza Nascimento, Raul Silva de Avellar, Beatriz Brilhante de Sousa, Artur de Barros Vaz Nascimento, João Guilherme Juarez Peres, Paula Izabelle Pantoja Veloso, Débora Elem Cruz Monteiro, Luis Felipe Pantoja Siqueira, Moisés Hamoy

**Affiliations:** Laboratory of Pharmacology and, Toxicology of Natural Products, Federal University of Pará (UFPA), R. Augusto Corrêa, Guamá, Belém, 01 - 66075-110 Brazil

**Keywords:** Anesthesia, Electrocardiogram, Opercular recording, Teleost, Posture reflex, Tambaqui

## Abstract

The search for safe and effective anaesthetic agents for aquaculture is essential to improve fish welfare and management practices. This study evaluated the anaesthetic efficacy of *Origanum vulgare* essential oil (OVEO) in juvenile tambaqui (*Colossoma macropomum*), an important Amazonian species. A total of 108 fish were exposed to immersion baths with increasing OVEO concentrations (19.04–57.12 mg L⁻^1^). Behavioral endpoints, electrocardiographic activity, opercular movements, and plasma glucose were assessed to determine induction, maintenance, and recovery profiles. At 19.04–38.08 mg L⁻^1^, OVEO induced rapid loss of equilibrium with full recovery, establishing a safe anaesthetic window. Higher concentrations (47.60–57.12 mg.L⁻^1^) produced deep anesthesia but also concentration-dependent bradycardia, reduced opercular frequency, increased glycaemia, and prolonged recovery times. Electrophysiological analysis revealed a progressive reduction in heart rate (up to 48.5%) and prolongation of RR and PQ intervals, without QRS morphology alteration, indicating reversible sinus bradycardia rather than conduction block. Opercular electromyography also demonstrated concentration-dependent respiratory depression with complete reversibility at lower concentrations. These results provide the first detailed electrophysiological evidence of OVEO-induced anesthesia in *C. macropomum*, supporting its use as a natural, effective, and sustainable anaesthetic for Amazonian aquaculture, contributing to improved animal welfare and management strategies.

## Introduction

Anesthesia using essential oils has gained considerable visibility in the veterinary field as a safer way to perform this procedure and reduce the risks to fish undergoing anesthetic procedures, with eugenol being the main representative (Pramod et al. 2010; Taleuzzaman et al. 2021; Ulanowska & Olas 2021). However, there are several studies of essential oils as having a potential anesthetic effect in various fish species (Toni et al. 2014; Dos Santos et al. 2017; Khumpirapang et al. 2018; Ferreira et al. 2021; Moreira et al. 2024). Thus, oregano (*Origanum vulgare*) essential oil, rich in phenols, especially carvacrol and thymol, has had a significant scientific impact, as studies have demonstrated its potent antimicrobial (Li et al. 2025; Tao et al. 2025), antifungal (Stamova et al. 2025), antiviral (Gosh, Sanyal & Sharma, 2022), and antioxidant (Tejada-Muñoz et al. 2024) action, becoming the subject of research in both human health (as a natural alternative to antimicrobial resistance) and veterinary medicine and food preservation.

The synergism among the components of *Origanum vulgare* essential oil (OVEO), especially carvacrol, thymol, p-cymene, and γ-terpinene, enhances its biological activity beyond what each molecule alone could achieve. Plant-based essential oils have been increasingly explored as natural anesthetic agents in aquaculture due to their safety, biodegradability, and multiple bioactive constituents. Their main active compounds, such as carvacrol and thymol in *Origanum vulgare*, exhibit synergistic interactions that may enhance sedative and anesthetic efficacy, as previously observed in other plant-based anesthetics (Can et al. 2018, 2019). These synergistic mechanisms are thought to result from combined actions on neuronal ion channels and GABAergic modulation, producing safer and more efficient anesthesia compared to single-compound formulations (Ghit et al. 2021). Therefore, OVEO represents a promising candidate for fish anesthesia; however, its anesthetic activity remains little explored (Capatina et al. 2021; Gabriel et al. 2022; Bodur, Oktavia & Sulmartiwi, 2024), essential oilparticularly in Amazonian fish species with zootechnical potential, such as *Colossoma macropomum*, as a new alternative for sedation and anesthetic induction.

During anesthesia in fish, neurophysiological and cardiovascular changes are observed and can be monitored through electrophysiological techniques such as electrocardiogram, electromyogram, and opercular movement recording (Fujimoto et al. 2017; Cantanhêde et al. 2020; Dos Santos et al. 2024; da Paz et al. 2024, 2025; Lins et al. 2025; Reis et al. 2025).

This study presents the first electrophysiological characterization of the anesthetic effects of OVEO in *Colossoma macropomum*, integrating electrocardiographic and opercular electromyographic analyses to establish a safe and reversible anesthetic window. It also provides quantitative evidence of cardiac modulation, demonstrating a concentration-dependent reduction. Furthermore, it is the first to associate opercular electromyography with anesthetic depth in this species. Together, these findings offer a new electrophysiological framework for evaluating anesthetic safety in Amazonian fish, advancing both animal welfare monitoring and the pharmacophysiological understanding of plant-derived anesthetics. Therefore, the present study aimed to use different concentrations of *Origanum vulgare* oil to evaluate safe anesthetic concentrations for the induction of superficial and deep anesthetic planes during induction and recovery, through electrophysiological monitoring and behavioral and glycemic assessments in *Colossoma macropomum*.

## Material and methods

### Organoleptic properties and chromatographic analysis

**Fig. 1:**
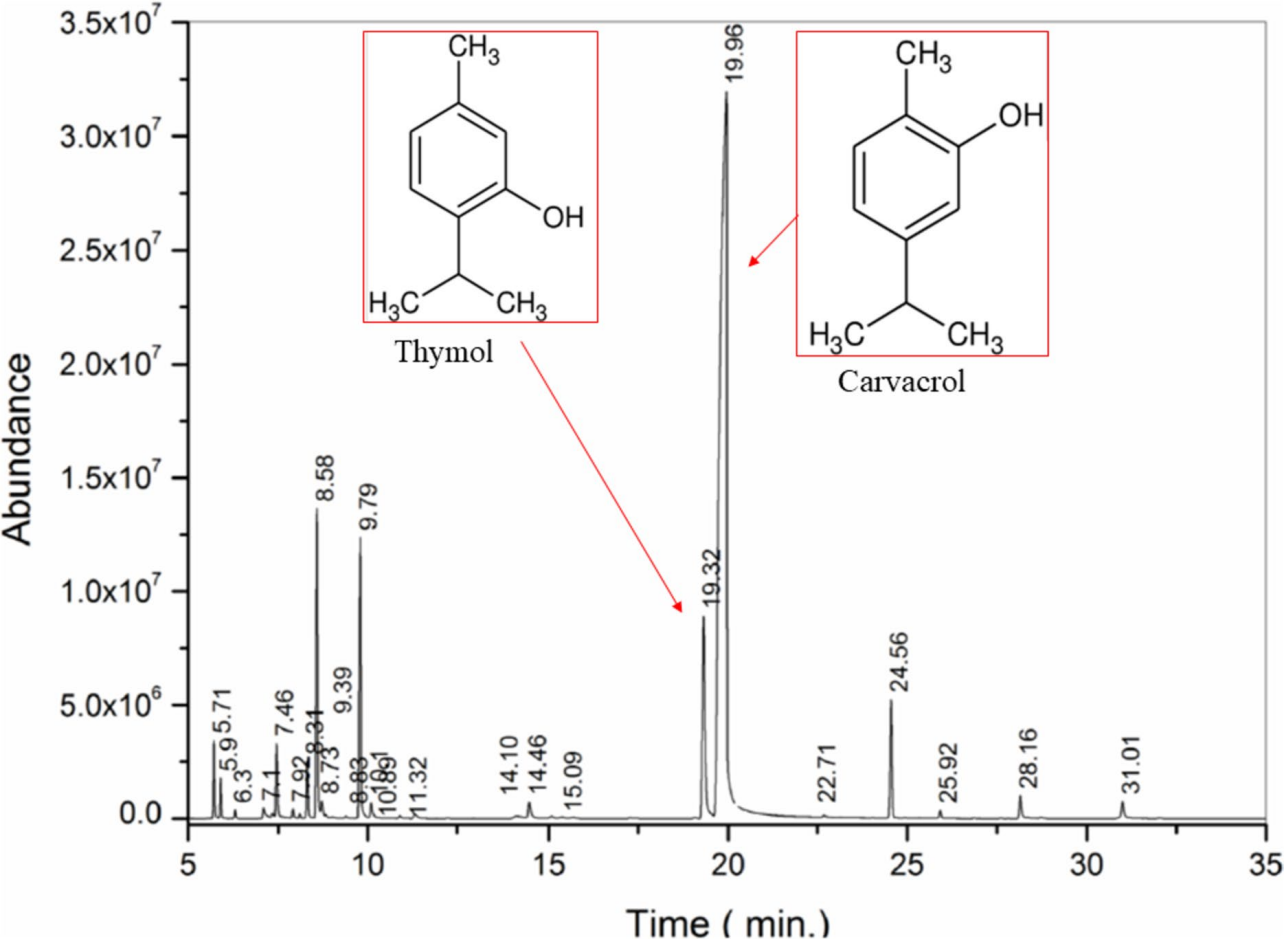
Chromatogram of the OVEO sample, gas chromatography – GC–MS mass spectrum. Department of Chemical Engineering and Food Engineering (EQA), Technology Center (CTC), Federal University of Santa Catarina – Brazil. Date of analysis 227.09.2023. Sample registration number 23/094. Major substances highlighted

OVEO was obtained from the Harmonie Laboratory (Registration: 11.938.821/0001–90), extracted by distillation and steam distillation, with a density of 0.952 g/ml and analyzed by the Department of Chemical Engineering and Food Engineering (EQA), Technology Center (CTC), Federal University of Santa Catarina under sample registration 23/094. The analysis date was September 27, 2023. The analysis method was High Performance Gas Chromatography on an AGILENT 7820 A Gas Chromatograph under the following conditions: Column: HP-5 30 m × 0.32 mm × 0.25 μm (AGILENT). Temp.: Column: 70 °C (0 min), 3 °C/min at 250 °C. Injector: 250 °C Division: 1/50. FID detector: 260 °C. Injection vol.: 1 µl (1% in chloroform). The phytoconstituents that compose the oil are described in Table 1, with the major components being Carvacrol (67.49%) and Thymol (7.25%). It has a purity level of 100% and, as it is an oleaginous substance, it was diluted with 70% alcohol in the proportion of 1 mL of oil/9 mL of alcohol and stored in a refrigerator at 4 °C for later withdrawal of an aliquot for the experiment (Fig. 1).

**Table 1:** Phytoconstituents that compose the OVEO

Retention Time	Substance Identification	%
5.71	βThuiene	1.21
5.59	Α- Pinene	0.63
6.31	Camphene	0.14
7.10	β-Pinene	0.37
7.35	3-Octanene	0.15
7.46	Myrcene	1.56
7.92	α -Phellandrene	0.20
8.11	3-Carene	0.09
8.31	α -Terpinene	1.08
8.59	p -Cymene	6.32
8.73	D-Limonene	0.50
8.83	Euclyptol	0.10
9.39	β- Ocimene	0.06
9.79	γ- Terpinene	6.00
10.10	Cis-Sabinene hydrate	0.50
10.89	α-Terpinolene	0.08
11.32	Trans-Sabinene hydrate	0.20
14.10	Borneol	0.24
14.46	Terpinen-4-ol	0.55
15.09	α-Terpineol	0.08
15.38	Dihydrocarvone	0.07
19.32	Thymol	7.25
19.95	Carvacrol	67.49
22.68	Carvacrol acetate	0.08
24.56	Caryphyllene	2.96
25.92	Humulene	0.20
28.15	β-Bisabolene	0.66
31.01	Caryophyllene oxide	0.66
	Minority compounds (< 0.06%)	0.26
	Unidentified compounds	0.35

### Source of animals

The animals were kept in 200-L aquariums in the Experimental Vivarium of the Laboratory of Pharmacology and Toxicology of Natural Products at the Federal University of Pará.

#### Experimental animals

For the experiments, 108 *Colossoma macropomum* were used. The system included water recycling for oxygenation, an ambient temperature of 26.3 °C, and a 12-h photoperiod. The water was renewed daily with 30% of the volume using water from the same source. Feeding was provided twice daily with commercial feed (32% protein), followed by a siphoning procedure to remove uneaten food and feces. During acclimation (10 days), water quality variables such as water temperature (26.3 °C) and hydrogen potential (pH) (pH = 7.53) were monitored.

#### Experimental design

Experiment with *Origanum vulgare* Essential Oil.

The tambaqui juveniles (16.30 ± 3.1 g) were randomly assigned to the following treatments: a) Control; b) Vehicle group (fish subjected to an immersion bath with 1.0 mL of 70% alcohol diluted in 1 L of aquarium water, not replicated tanks were used); c) Fish treated with OVEO 19.04 mg. L^−1^; d) 28.56 mg. L^−1^; e) 38.08 mg. L^−1^; f) 47.60 mg. L^−1^; g) 57.12 mg. L^−1^. All fish underwent anesthetic induction by maintaining contact for a period of 5 min, and for anesthetic recovery, they were observed for 5 min in water without OVEO. For each recording, n = 9/treatment (immersion bath with OVEO and recovery after immersion bath) were used according to Can et al. (2018).

#### Experiment 1—Analysis of the characteristic behaviors of anesthetic induction and recovery and plasma glucose measurement 30 min after the anesthetic procedure

**Fig. 2:**
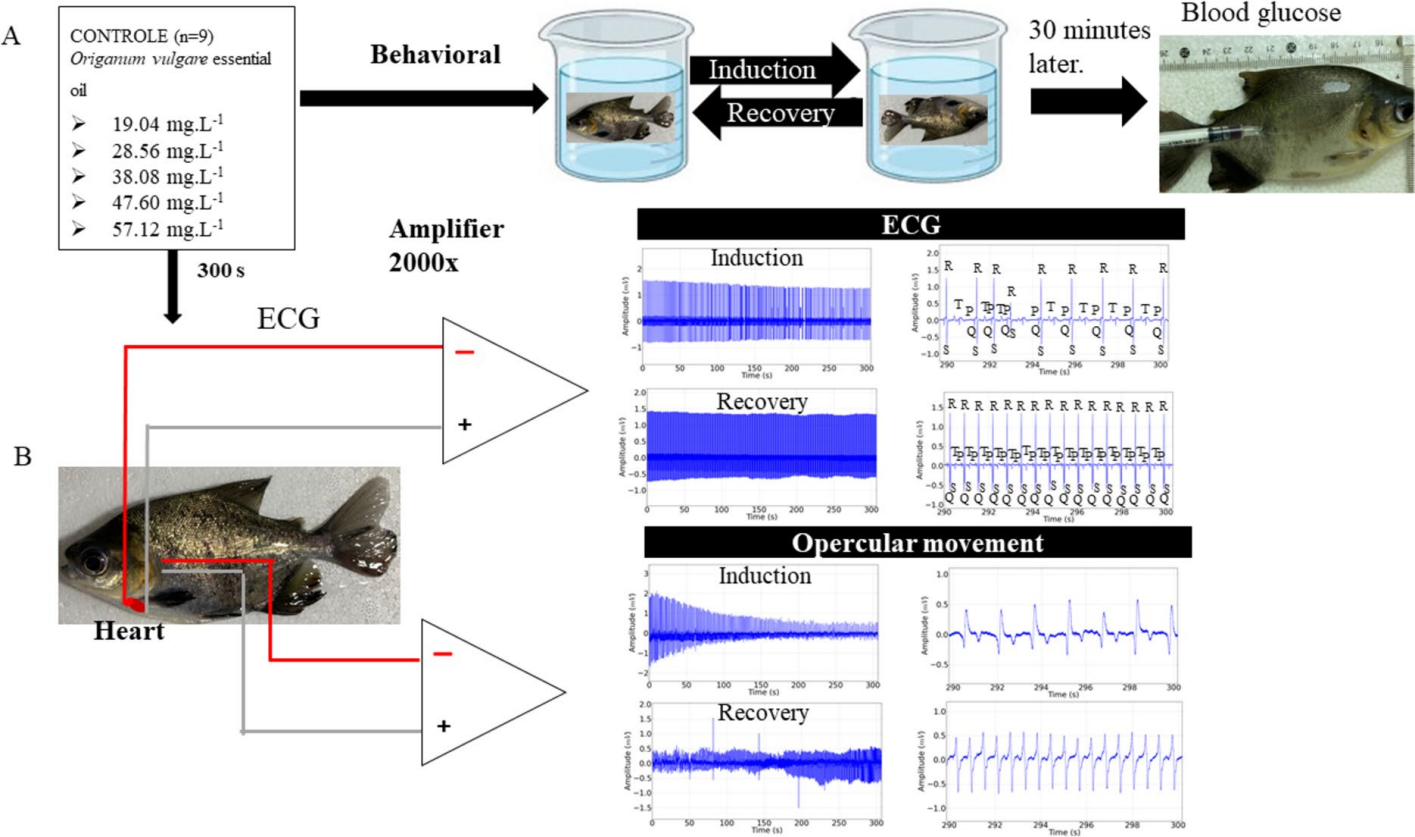
Diagram demonstrating the experimental design: Different concentrations of OVEO during anesthetic induction and recovery (latency) and blood glucose after 30 min of contact (**A**); Records of electrocardiographic activity (ECG) and recording of the opercular beat during anesthetic induction and recovery (**B**)

The latency for loss of the posture reflex and immobility, characterized by lateral recumbency and loss of somatic activity (anesthetic induction), were evaluated. Subsequently, the animals were removed from contact with OVEO, where the latency for recovery of the posture reflex was evaluated. Considering the contact time for the OVEO treatments: a) 19.04 mg. L^−1^, b) 28.56 mg. L^−1^, c) 38.08 mg. L^−1^, d) 47.60 mg. L^−1^, and e) 57.12 mg. L^−1^ (n = 9/treatment), for a total of 45 animals (Fig. 2A). Thirty minutes after contact with OVEO, blood was collected from the lateral tail vein for blood glucose analysis using an Accu-Chek Active® glucometer.

#### Experiment 2—Electrocardiographic (ECG) recordings and opercular activity during anesthetic induction and recovery

To analyze cardiac function recordings, 925 silver electrodes with a diameter of 0.3 mm and a length of 12 mm were made and subsequently insulated with a liquid insulator (Quimatic®). The electrodes were manufactured in a non-conjugated manner. The reference electrode was position a indicating the cardiac vector (negative pole at the base of the heart and positive pole at the apex of the heart), and was attached to the ventral portion of the left operculum 0.2 mm before the end of the opercular cavity. The recording electrode was inserted 2.0 mm before the end of the right opercular opening. The electrodes captured the signal near the D1 lead. The electrodes were connected to a high-impedance amplifier (Grass Technologies, Model P511) to acquire electrocardiographic recordings. This enabled the analysis of heart rate (bpm), QRS complex amplitude (mV), QRS complex duration (ms), R-R (ms), P-Q (ms), and S-T (ms) intervals (Fig. 2B).

To record opercular activity, 925 silver electrodes with a diameter of 0.5 mm and a length of 20 mm were made. The electrodes were spaced 7 mm apart and insulated with liquid insulation (Quimatic®). The electrode attachment position for recording the opercular beat was the central part of the right opercular aperture. During recording, the frequency (opercular movements per minute) and the power of the opercular beat (mV2/Hz) were assessed (Fig. 2B).

To acquire the recording signals, the groups were divided as follows: a) Control group; b) Vehicle group; c) Group treated with OVEO 19.04 mg. L^−1^; d) 28.56 mg. L^−1^; e) 38.08 mg. L^−1^, f) 47.60 mg. L^−1^ and g) 57.12 mg. L^−1^ (*n* = 9, total of 63 animals).

#### Recording and analysis

Data were analyzed using a tool built in the Python programming language. The Numpy and Scipy libraries were used for mathematical processing, and the Matplolib library was used for graphing. The graphical interface was developed using the PyQt4 library (Reis et al. 2025).

The electrodes were connected to a digital data acquisition system through a high-input impedance differential amplifier (Grass Technologies, Model P511), set to 0.3 and 300 Hz filtering, with 2000X amplification, and monitored with an oscilloscope (ProteK, Model 6510). Recordings were continuously digitized at a rate of 1 kHz on a computer equipped with a data acquisition card (National Instruments, Austin, TX). They were stored on a hard drive and later processed using specialized software (LabVIEW express).

### Statistical analysis

After assessing compliance with the assumptions of normality and homogeneity of variances using the Kolmogorov–Smirnov and Levene tests, respectively, comparisons of mean power values ​​were made using two-way ANOVA followed by Tukey's test. GraphPad Prism® 8 software was used for the analyses, and values ​​of *p < 0.05, **p < 0.01, and ***p < 0.001 were considered statistically significant in all cases.

## Results

**Fig. 3:**
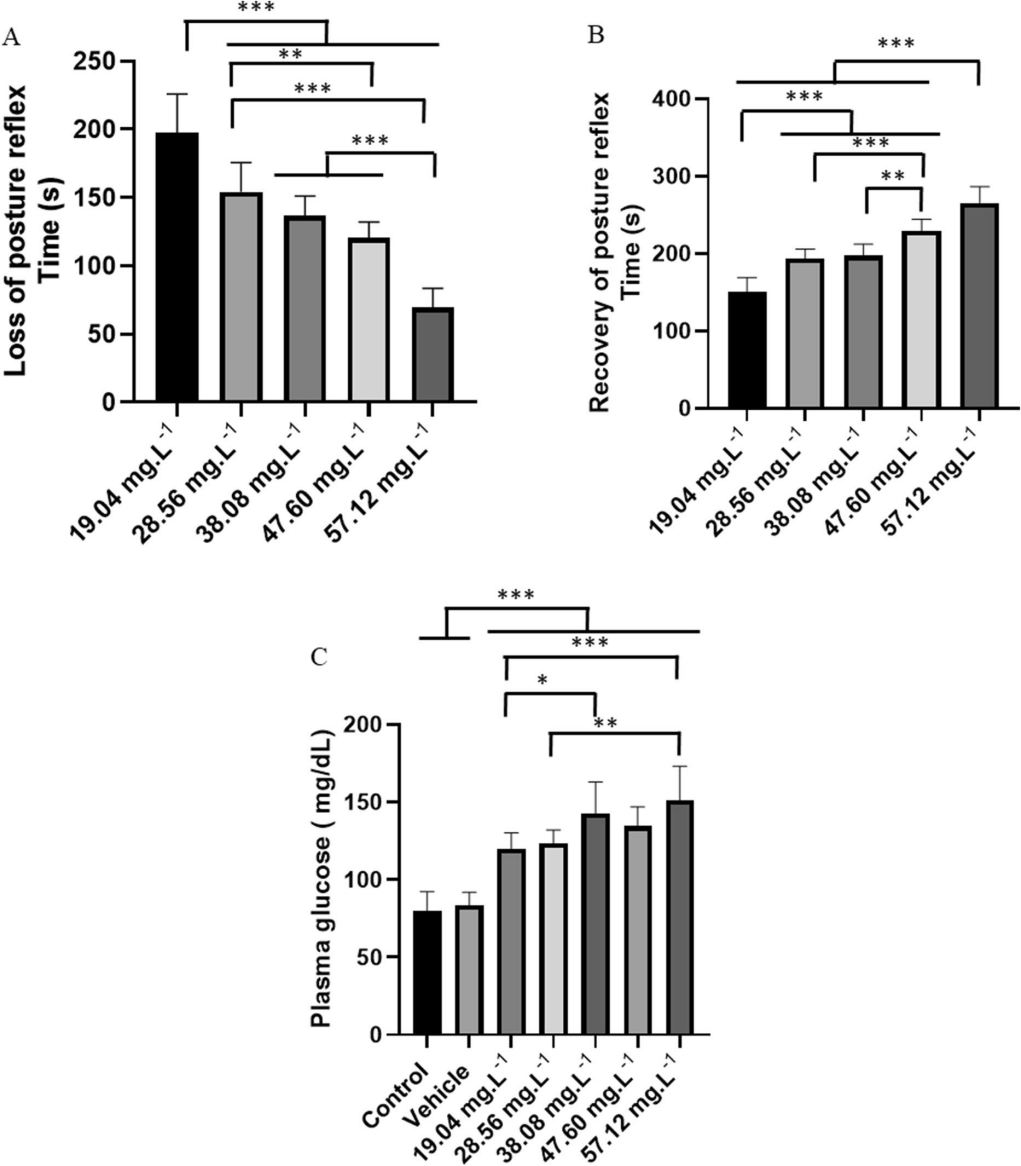
Mean latencies in seconds during the immersion bath for loss of the postural reflex in different OVEO treatments (**A**); Recovery of the postural reflex after immersion bath with OVEO (**B**); Plasma blood glucose measured after 30 min of treatment (**C**). (ANOVA followed by Tukey's test; *p < 0.05, **p < 0.01 and ***p < 0.001)

Treatment with OVEO demonstrated loss of postural reflex that progressed to immobility, with decreased latency to the onset of behavior in treatments with higher concentrations. Fish treated with 19.04 mg L⁻^1^ showed lower latency than the other treated groups. The group treated with 28.56 mg L⁻^1^ showed similar performance to the group treated with 38.08 mg L⁻^1^ (p = 0.299), but inferior to the other groups. The group treated with 38.08 mg L⁻^1^ showed similar performance to the group treated with 47.06 mg L⁻^1^ (p = 0.370), but superior to the group treated with 57.12 mg L⁻^1^ (Fig. 3A).

The recovery of the postural reflex in the group treated with 19.04 mg. L^−1^ of OVEO had a mean latency of 150.9 ± 18.19 s, which was inferior to the other groups. The group treated with 28.56 mg. L^−1^ (193.0 ± 12.76 s), was similar to the group treated with 38.08 mg. L^−1^(p = 0.986) but were lower than the other groups. All treated groups were superior to the group treated with 57.12 mg. L^−1^ (265.6 ± 12.76 s) (Fig. 3B).

For plasma glucose, the control group presented a mean of 79.89 ± 12.33 mg/dL, similar to the vehicle group (p = 0.998), but lower than the other groups. The group treated with 19.04 mg/L (120 ± 10.19 mg/dL) presented values ​​similar to the groups treated with 28.56 mg/L (p = 0.999) and 47.60 mg/L (p = 0.354). The group treated with 57.12 mg/L (151 ± 10.19 mg/dL) presented values ​​similar to the groups treated with 38.08 mg/L (p = 0.8816) and 47.60 mg/L (p = 0.204) (Fig. 3C).

**Fig. 4:**
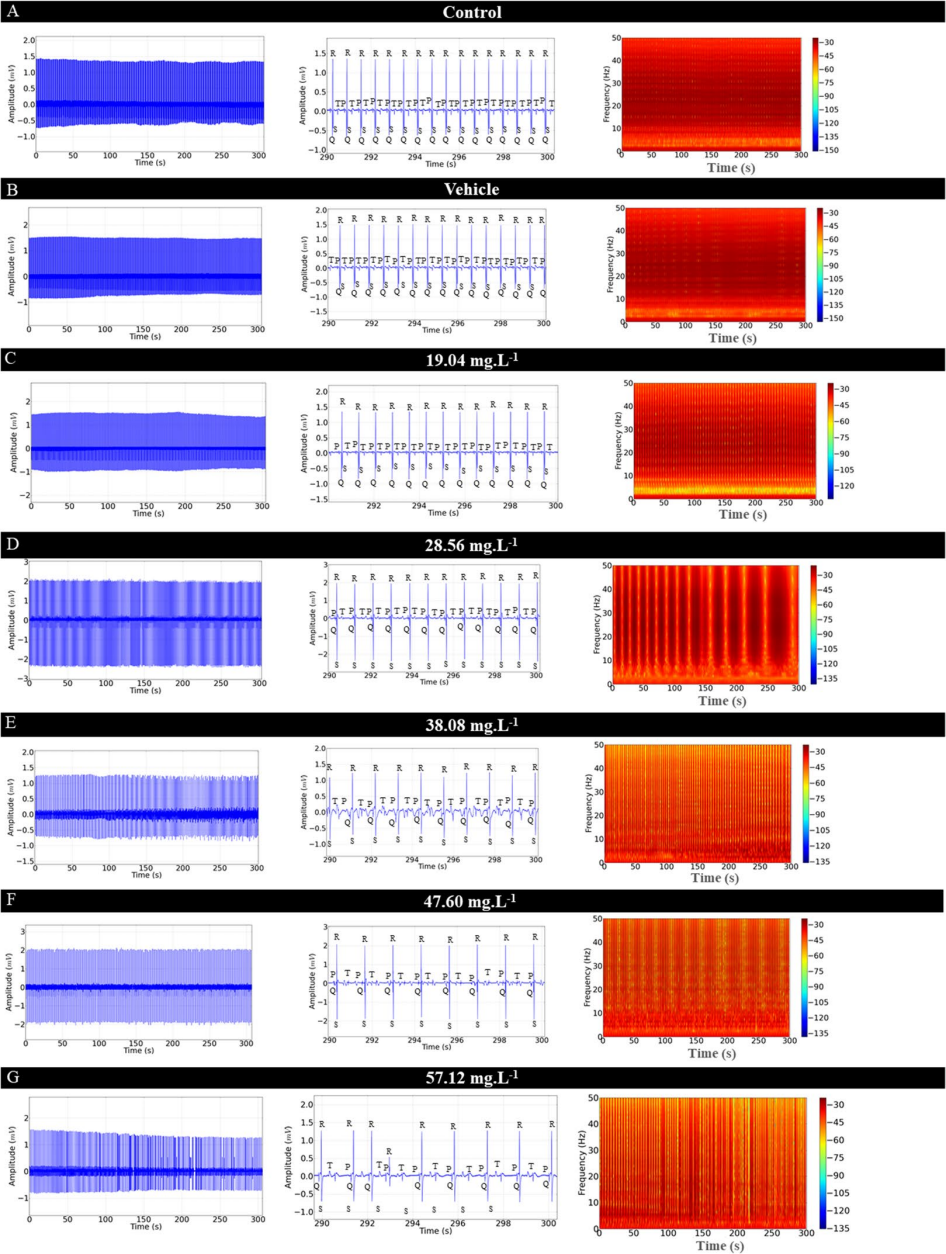

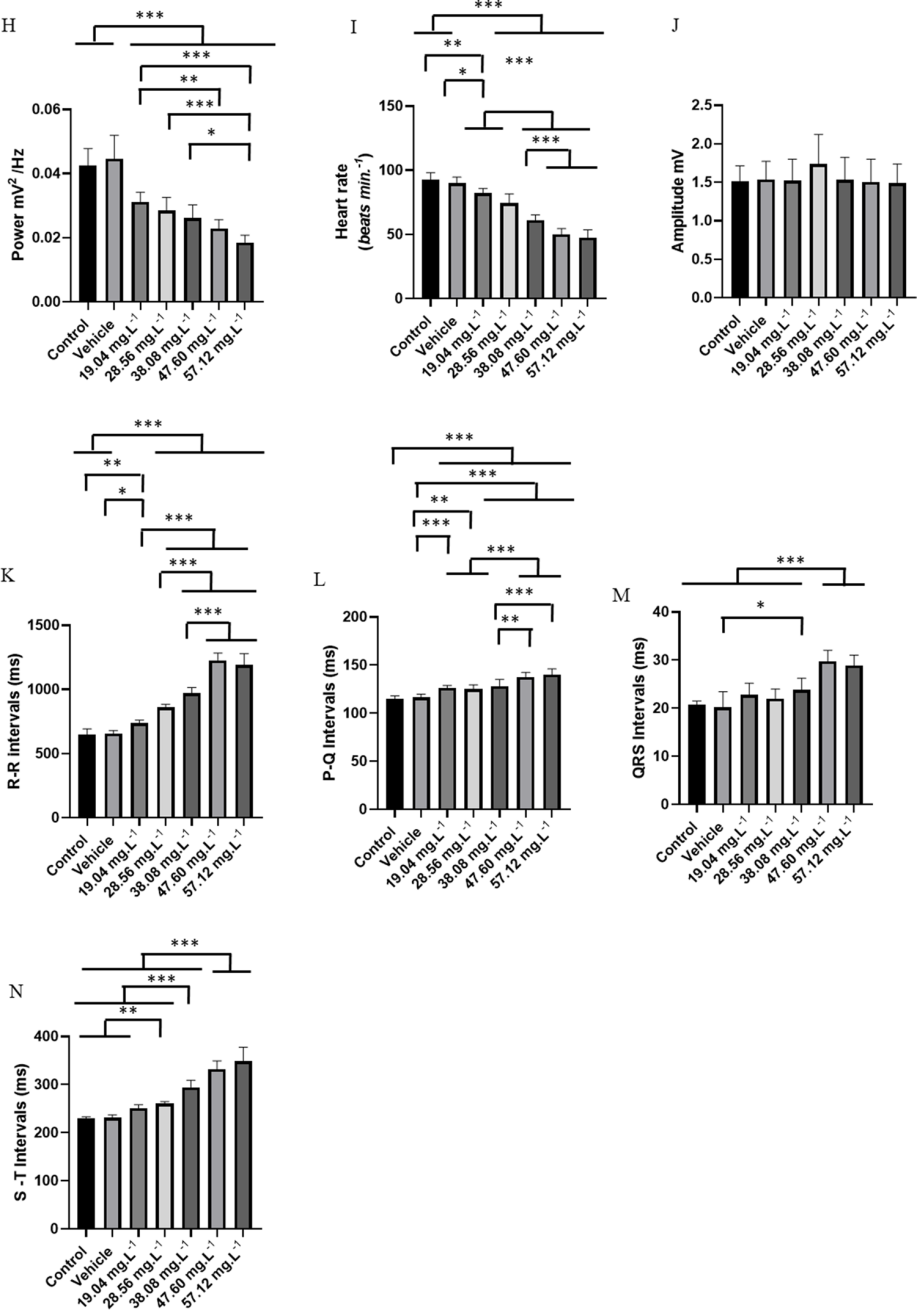
Electrocardiographic recordings demonstrating cardiac activity in juvenile Colossoma macopomum (left), amplification of the 10 s at the end of the 5-min recording (290—300 s), with demonstration of characteristic morphographic structures and electrocardiogram such as the P wave, QRS complex and T wave (center) and spectrograms demonstrating the power distribution during the ECG recording (left), for the following groups: Control group (**A**), Vehicle group (**B**), group treated with 19.04 mg. L^−1^ (**C**), 28.56 mg. L^−1^ (**D**), 38.08 mg. L^−1^ (**E**), 47.60 mg. L^−1^ (**F**), 57.12 mg. L.^−1^ (**G**). Analysis of data shown in linear power graphs of cardiac recordings mV2/Hz (**H**), Heart rate (bpm) (**I**), QRS complex amplitude (mV) (**J**), RR interval (ms) (**K**), PQ interval (ms) (**L**), QRS duration (ms) (**M**), ST interval (ms) (**N**). (ANOVA followed by Tukey's test; *P < 0.05, **p < 0.01, ***p < 0.001; *n* = 9)

The ECG of *C. macropomum* for the control group showed sinus rhythm and a mean frequency of 92.44 ± 5.63 bpm. The graphoelements were identified as the P wave, the QRS complex, and the T wave (Fig. 4A). Cardiac activity in the control group was similar to the vehicle group (p = 0.955) (Fig. 4B). From the 10-s amplification, the intervals were evaluated during treatment with OVEO in an immersion bath (Fig. 4C, D, E, F, and G).

During treatment in an immersion bath with 19.04 mg. L^−1^, the animals showed a reduction in heart rate of 11.29% compared to the control group (Fig. 4C). The group treated with 28.56 mg. L^−1^ of OVEO showed a decrease of 19.47% (Fig. 4D). The group treated with 38.08 mg. L^−1^ showed a decrease of 33.89% (Fig. 4E). The group treated with 47.60 mg. L^−1^ had a reduction of 45.91% (Fig. 4F), and the group treated with 58.12 mg. L^−1^ showed a decrease of 48.55% (Fig. 4G). The treated fish presented sinus bradycardia, which was intensified by the increase in concentration. The decrease in heart rate decreases the energy level recorded in the spectrograms (Fig. 4A, B C, D, E, F and G).

The mean linear power during recordings in the treatments indicates a decrease in power in the spectrograms, thus, for the control group the mean power was 0.0425 ± 0.005 mV2/Hz was similar to the vehicle group (p = 0.954), however, they were higher than the other treated groups. The groups treated with 19.04 mg. L^−1^ (0.031 ± 0.003 mV^2^/Hz) was similar to the groups treated with 28.56 mg. L^−1^ (p = 0.863) and 38.08 mg. L^−1^ (p = 0.235). The mean of the group treated with 28.56 mg. L^−1^ (0.028 ± 0.0041 mV^2^/Hz) was similar to the groups treated with 38.08 mg. L^−1^ (p = 0.928) and 47.60 mg. L^−1^ (p = 0.129), but was superior to the group treated with 57.12 mg. L^−1^) group treated with 57.12 mg. L^−1^ (0.0185 ± 0.0023 mV^2^/Hz) was similar to the group treated with 547.60 mg. L^−1^ (p = 0.380) (Fig. 4 H).

Heart rate decreased during treatment with increasing concentrations of OVEO. The control group had a mean of 92.44 ± 5.63 bpm, similar to the vehicle group (p = 0.955), but was higher than the other groups. The group treated with 19.04 mg. L^−1^ had a mean of (82.00 ± 3.87 bpm), similar to the group treated with 28.56 mg. L^−1^ (p = 0.0522), but was higher than the other groups. The group treated with 57.12 mg. L^−1^ (47.56 ± 5.98 bpm) was similar to the group treated with 47.60 mg. L^−1^ (p = 0.955), but was lower than the group treated with 38.08 mg. L^−1^ (Fig. 4 I).

The mean QRS complex amplitude for the control group was 1.50 ± 0.204 mV and was similar for the vehicle group and all groups treated with OVEO (F (6, 56) = 0.844; p = 0.540) (Fig. 4J).

The mean RR interval for the control group was 650.4 ± 42.65 ms, similar to the mean RR interval for the vehicle group (p = 0.999), but was shorter than the other groups. The group treated with 19.04 mg. L^−1^ (738.0 ± 24.02 ms) was shorter than the other treated groups. The group treated with 28.56 mg. L^−1^ (859.7 ± 25.02 ms) was shorter than the groups treated with higher concentrations. The groups treated with 47.60 mg. L^−1^ (228 ± 56.52 ms) were similar to the group treated with 57.12 mg. L^−1^ (p = 0.689) however, they were superior to the group treated with 38,08 mg. L^−1^ (969 ± 46.73 ms) (Fig. 4K).

The mean PQ interval for the control group was 115.1 ± 3.01 ms, similar to the vehicle group (p = 0.997), but lower than the other treated groups. The group treated with 19.04 mg. L^−1^ (126.4 ± 2.50 ms) was similar to the groups treated with 28.56 mg. L^−1^ (p = 0.996) and 38.08 mg. L^−1^ (p = 0.987). The group treated with 47.60 mg. L^−1^ (137.7 ± 4.77 ms) was similar to the group treated with 57.12 mg. L^−1^ (p = 0.904), but higher than the other groups (Fig. 4L). The mean QRS complex duration for the control group (20.67 ± 0.86 ms) was similar to the vehicle (p = 0.999), 19.04 mg. L^−1^ (p = 0.460), 28.56 mg. L^−1^ (p = 0.880), and 38.08 mg. L^−1^ (p = 0.080) groups. However, they were lower than the 47.60 mg. L^−1^ and 57.12 mg. L^−1^ treated groups. The 47.60 mg. L^−1^ treated group (29.78 ± 2.27 ms) was similar to the 57.12 mg. L^−1^ treated group (p = 0.982) (Fig. 4M).

For the control group, the mean ST interval was 230.3 ± 2.82 ms, which was similar to the vehicle group (p = 0.999) and the group treated with 19.04 mg. L^−1^ (p = 0.065), but was lower than the other treated groups. The group treated with 19.04 mg. L^−1^ (250.8 ± 7.49 ms) was similar to the group treated with 28.56 mg. L^−1^ (p = 0.785), however, they were lower than the other treated groups. The group treated with 47.60 mg. L^−1^ (331.9 ± 29.45 ms) was similar to the group treated with 57.12 mg. L^−1^ (p = 0.230), being higher than the other groups (Fig. 4 N).

**Fig. 5:**
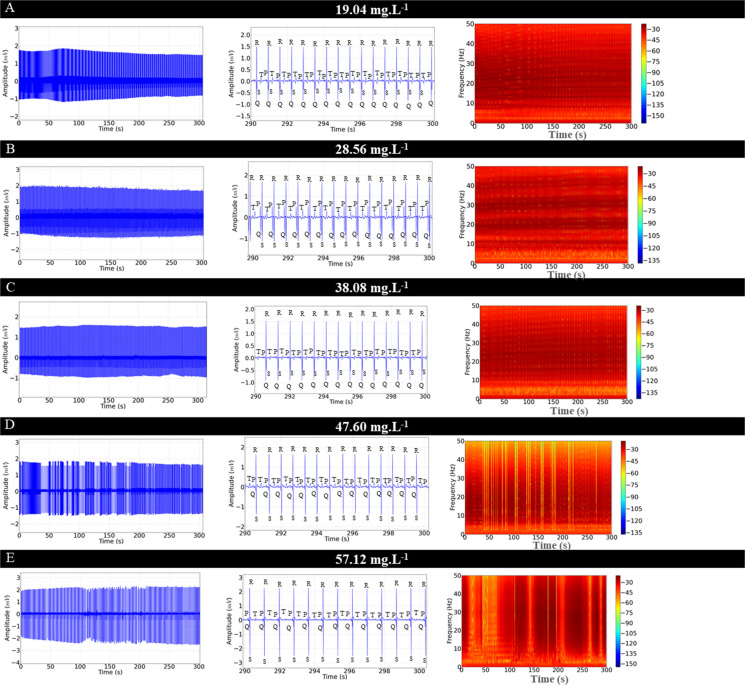

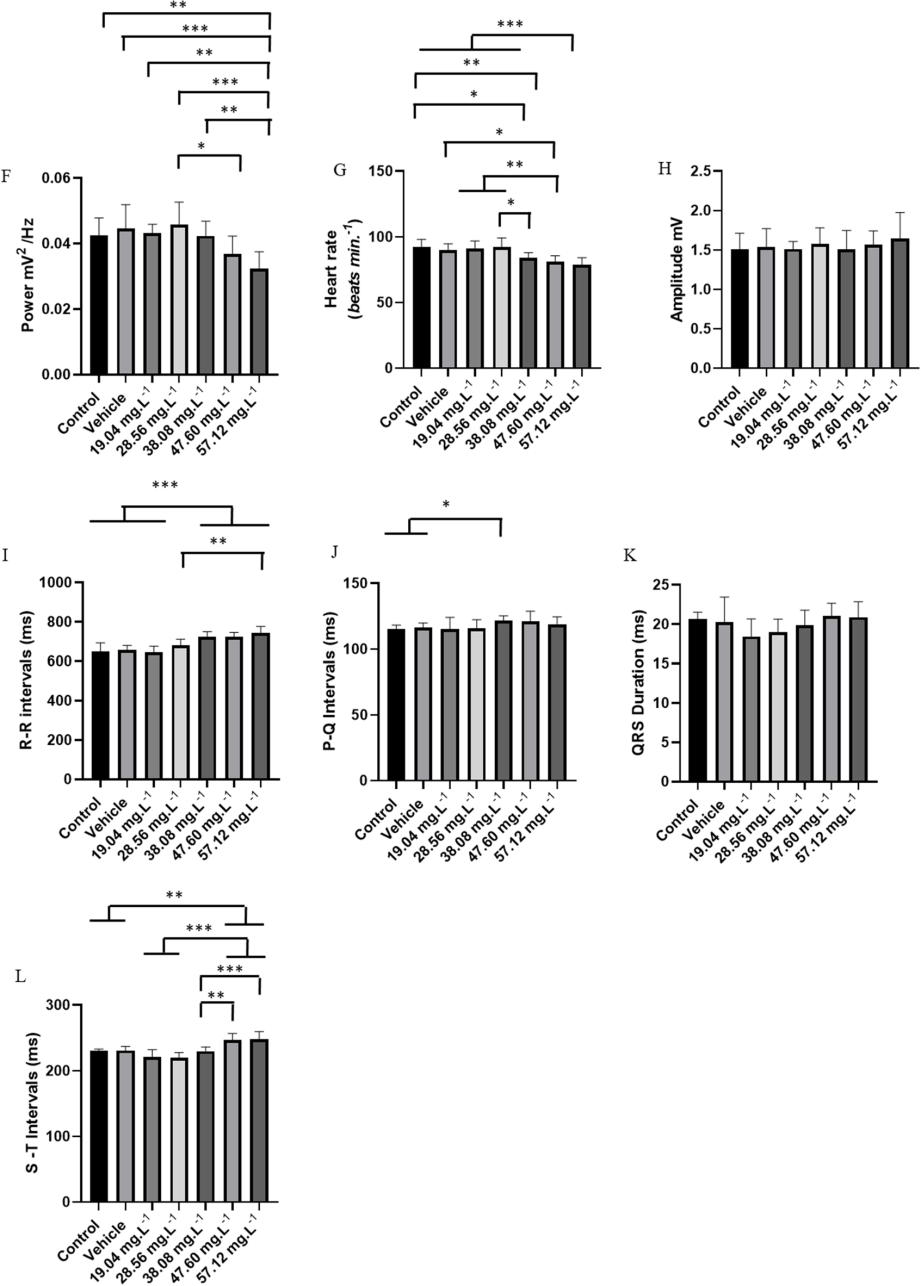
Cardiac evaluation in juvenile *Colossoma macropomum*, after treatment in an immersion bath (left). Amplification of the recording in the last 10 s (290–300 s), with identification of cardiac triggers (center), Spectogram of energy distribution up to 50 Hz during the recovery period (right) for the groups treated with the following concentrations of OVEO: 19.04 mg. L^−1^ (A), 28.56 mg. L^−1^ (B), 38.08 mg. L^−1^ (C), 47.60 mg. L^−1^ (D) and 57.12 mg. L.^−1^ (E). Graphs demonstrating the average cardiac parameters during treatment recovery: Linear power during the recovery period mV2/Hz (F), Heart rate (bpm) (G), QRS amplitude (mV) (H), R-R interval (ms) (I), P-Q intervals (ms) (J); QRS complex duration (ms) (K) and ST interval (ms) (L). (ANOVA followed by Tukey's test; *P < 0.05, **p < 0.01, ***p < 0.001; *n* = 9)

During recovery from OVEO-induced anesthesia, reversibility of electrocardiographic changes was observed (Figs. 5A, B, C, D, and E). However, reversibility of the effects occurred slowly for the groups treated with higher concentrations.

Heart rate recovery in the group treated with 19.04 mg. L^−1^ compared to the control group was 98.44% (Fig. 5A). Fish treated with 28.56 mg. L^−1^ showed 99.52% heart rate recovery compared to the control groups (Fig. 5B). The group treated with 38.08 mg. L^−1^ of OVEO showed 90.86% (Fig. 5C), the group treated with 47.60 mg. L^−1^ showed 87.98% (Fig. 5D), and the group treated with 57.12 mg. L^−1^ showed 85.10% compared to the control group (Fig. 5E).

The mean linear power after treatments allowed us to identify reversibility based on the increase in power in the spectrograms. Thus, for the control group, the mean power was 0.0425 ± 0.005 mV2/Hz, similar to the vehicle group (p = 0.954) and groups treated with 19.04 mg. L^−1^ (p = 0.999), 28.56 mg. L^−1^ (p = 0.854), 38.08 mg. L^−1^ (p = 0.999), and 47.60 mg. L^−1^ (p = 0.294). The group treated with 57.12 mg. L-1 (0.0324 ± 0.005 mV2/Hz) was similar to the group treated with 47.60 mg. L^−1^ (p = 0.629), but was lower than the other groups (Fig. 5F). During the recovery period, the control group presented a mean heart rate of 92.44 ± 5.63 bpm, similar to the vehicle group (p = 0.960) and those treated with 19.04 mg. L^−1^ (p = 0.998) and 28.56 mg. L^−1^ (p = 0.999). However, it was superior to the group treated with 38.08 mg. L^−1^ (84.00 ± 4.00 bpm), 47.60 mg. L^−1^ (81.33 ± 4.35 ms) and 57.12 mg. L^−1^ (78.87 ± 5.47 ms). The group treated with 57.12 mg. L^−1^ (78.67 ± 5.47 bpm) was similar to the groups treated with 38.08 mg. L^−1^ (p = 0.369) and 47.60 mg. L^−1^ (p = 0.940) (Fig. 5G).

During recovery, the QRS complex amplitude in the control group showed a mean of 1.50 ± 0.20 mV, which was similar to the vehicle group (p = 0.999) and all treated groups during recovery from anesthesia (F (6, 56) = 0.474 p = 0.824) (Fig. 5 H).

The mean RR interval during recovery for the control group was 650.4 ± 42.65 ms, which was similar to the vehicle groups (p = 0.999), treated with 19.04 mg. L^−1^ (p = 0.999) and 28.56 mg. L^−1^ (p = 0.315), however, it was lower than the other treated groups. The group treated with 28.56 mg. L^−1^ (682.3 ± 29.58 ms) was similar to the groups treated with 38.08 mg. L^−1^ (p = 0.101) and 47.60 mg. L^−1^ (p = 0.078), however, it was lower than the group treated with 57.12 mg. L^−1^. The group treated with 38.08 mg. L^−1^ (722.6 ± 27.82 ms) was similar to the 47.60 mg. L^−1^ (p = 0.999) and 57.12 mg. L^−1^ (p = 0.800) groups (Fig. 5I).

The PQ interval during recovery in the control group was 115.1 ± 3.018 ms, similar to the vehicle group (p = 0.970), groups treated with 19.04 mg. L^−1^ (p = 0.999), 28.56 mg. L^−1^ (p = 0.999), 47.60 mg. L^−1^ (p = 0.208), and 57.12 mg. L^−1^ (p = 0.781). The group treated with 38.08 mg. L^−1^ (121.8 ± 3.49 ms) was superior to the control and vehicle groups (Fig. 5J).

During the recovery period, the mean QRS complex duration for the control group (20.67 ± 0.86 ms) was similar to that of the other groups (F (6, 56) = 2.055, p = 0.073) (Fig. 5K).

During the recovery period, the ST interval for the control group was 230.3 ± 2.82 ms, similar to that of the vehicle group (p = 0.999) and the groups treated with 19.04 mg. L^−1^ (p = 0.224), 28.56 mg. L^−1^ (p = 0.172), and 38.08 mg. L^−1^ (p = 0.999), but was inferior to the groups treated with 47.60 mg. L^−1^ and 57.12 mg. L^−1^. ​​The group treated with 47.60 mg. L^−1^ (246.3 ± 10.64 ms) was similar to the group treated with 57.12 mg. L^−1^ (p = 0.999) (Fig. 5L).

**Fig. 6:**
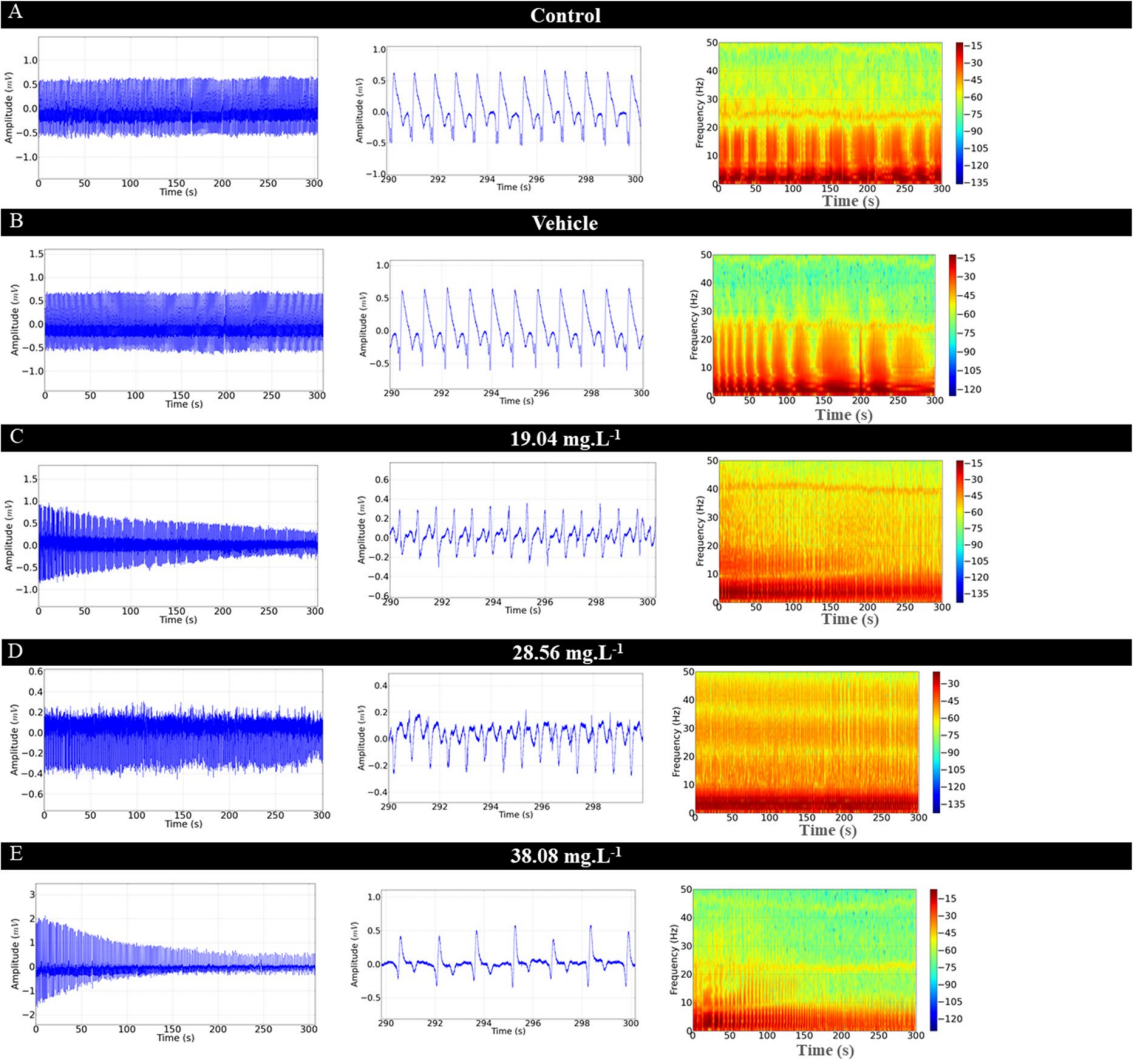

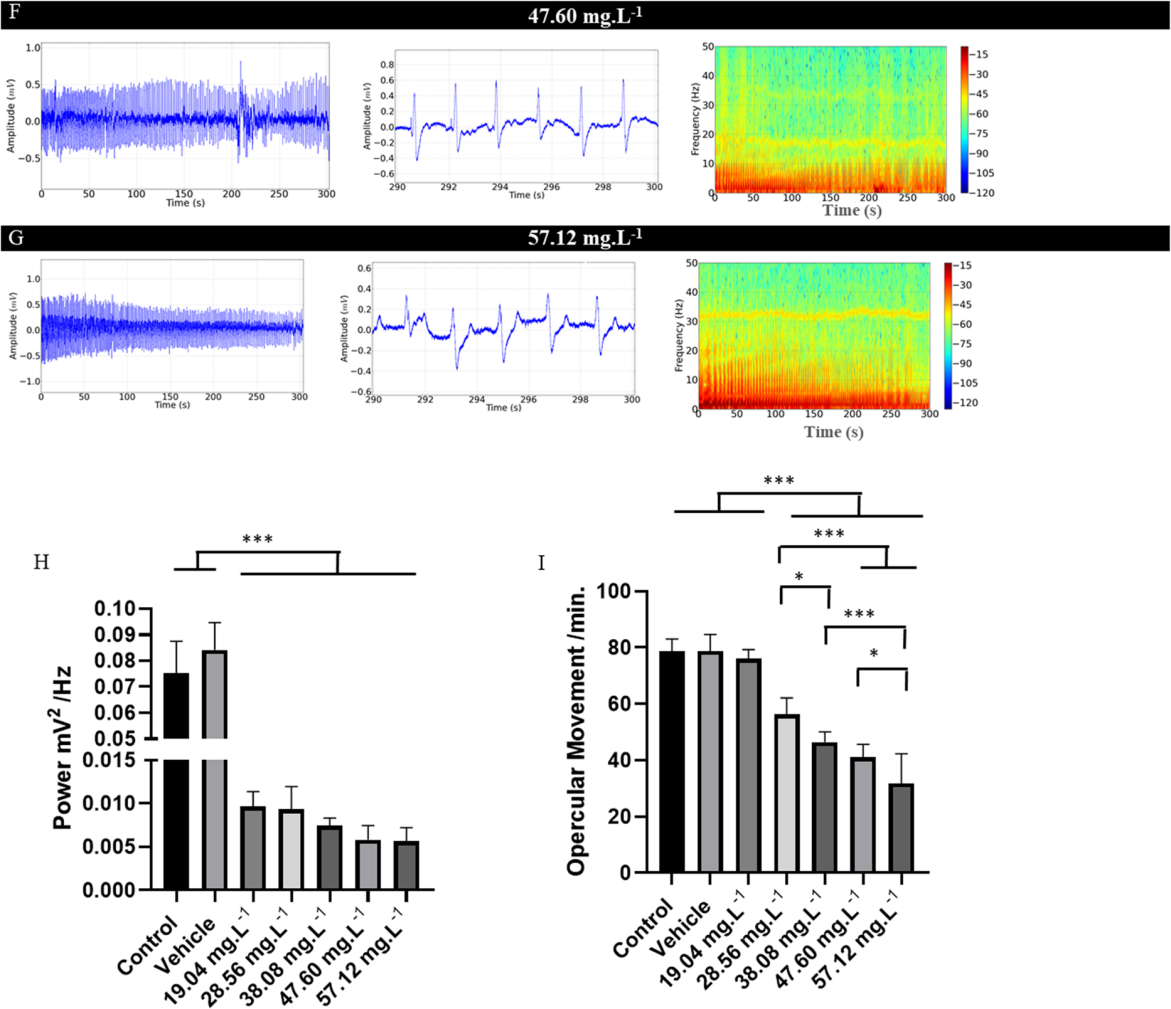
Recording of opercular activity of *Colossoma macropomum*, in an immersion bath with different concentrations of OVEO (left). Amplification of the recording in the last 10 s (290–300 s) (center), recording of the power distribution at a frequency of 50 Hz (right). Data obtained for the groups: Control group (A); Vehicle group (B); Group treated with 19.04 mg. L^−1^ (C); treatment with 28.56 mg. L^−1^ (D); treatment with 38.08 mg. L^−1^ (E), treatment with 47.60 mg. L^−1^ (F) and treatment with 57.12 mg. L.^−1^ (G). Graph showing the mean values ​​of opercular movement power (mV2/Hz) (H), mean values ​​of opercular movement per minute (I). (ANOVA followed by Tukey's test; *P < 0.05, **p < 0.01, ***p < 0.001; *n* = 9)

During treatment with OVEO at concentrations of 19.04 mg. L^−1^, 28.56 mg. L^−1^, 38.08 mg. L^−1^, 47.60 mg. L^−1^ and 57.12 mg. L^−1^ the frequency of opercular movement showed a concentration-dependent reduction when compared to the control group (Fig. 6A, B, C, D, E, F and G). The control group had a mean of 78.67 ± 4.35 movements per minute, which was similar to the vehicle group (p = 0.999) and the group treated with 19.04 mg. L^−1^ (p = 0.960), however, they were superior to the treated groups. The group treated with 28.56 mg. L^−1^ showed a decrease in the opercular beat in relation to the control group of 28.53%. The group treated with 38.08 mg. L^−1^ reduced (37.15%), the group treated with 47.60 mg. L^−1^ reduced (47.44%), and the group treated with 57.12 mg. L^−1^ showed a reduction of 59.60%. Thus, it can be observed that the energy level decreased in the spectrograms (Figs. 6A, B, C, D, E, F, and G).

There was a reduction in power in the recordings of opercular movements during anesthesia induction. The control group presented a mean power of 0.075 ± 0.012 mV2/Hz, which was similar to the vehicle group (p = 0.0517), but these groups were superior to the treated groups. All treated groups were similar (p = 0.833) (Fig. 5 H). The mean opercular movement for the control group was 78.67 ± 4.35 movements per minute, similar to the vehicle and 19.04 mg. L^−1^ groups (p = 0.0.960) but superior to the other treated groups. The groups treated with 38.08 mg. L^−1^ (56.22 ± 5.86 movements per minute) was similar to the group treated with 47.60 mg. L^−1^ (p = 0.479). All groups were superior to the group treated with 57.12 mg. L^−1^ (Fig. 6I).

**Fig. 7:**
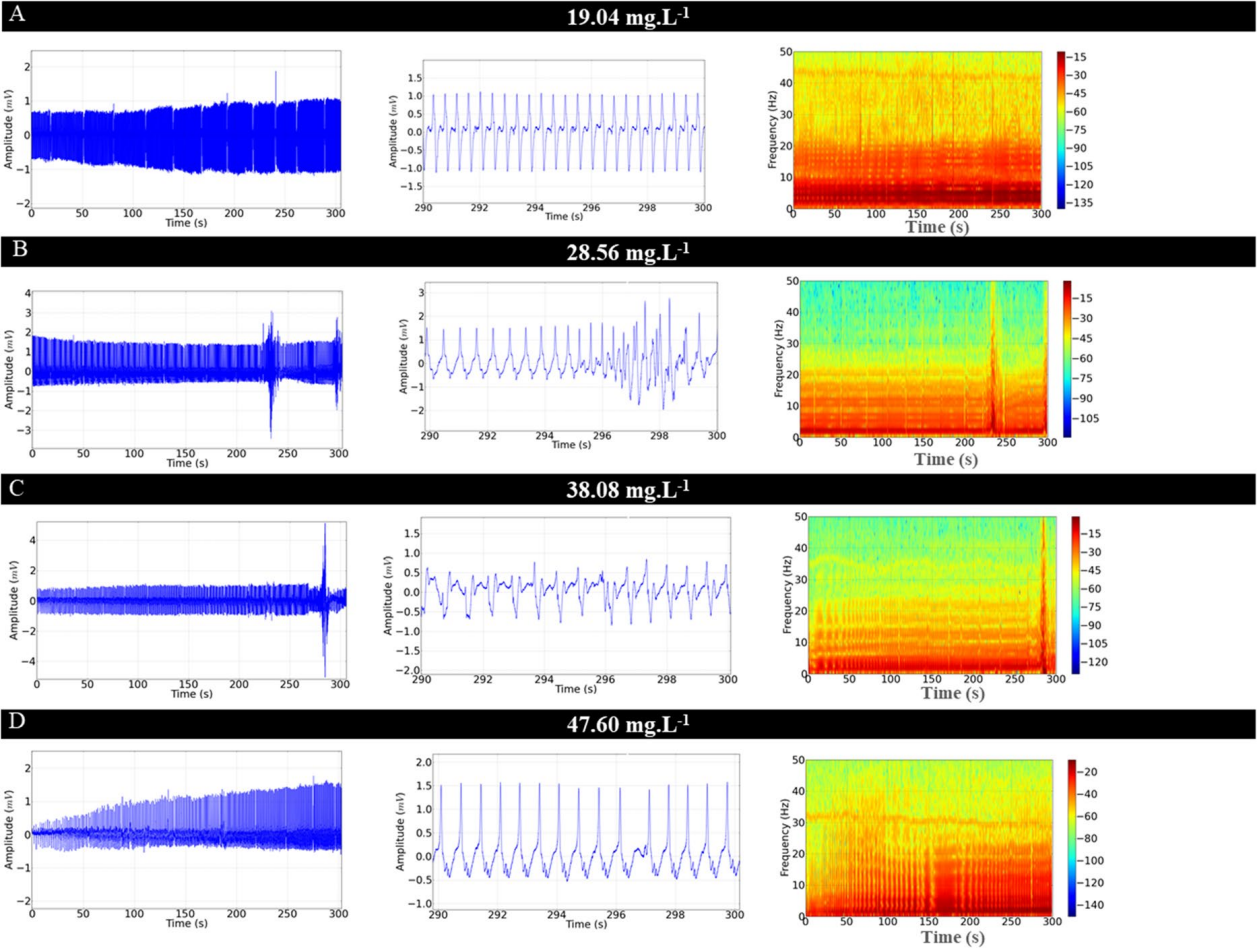

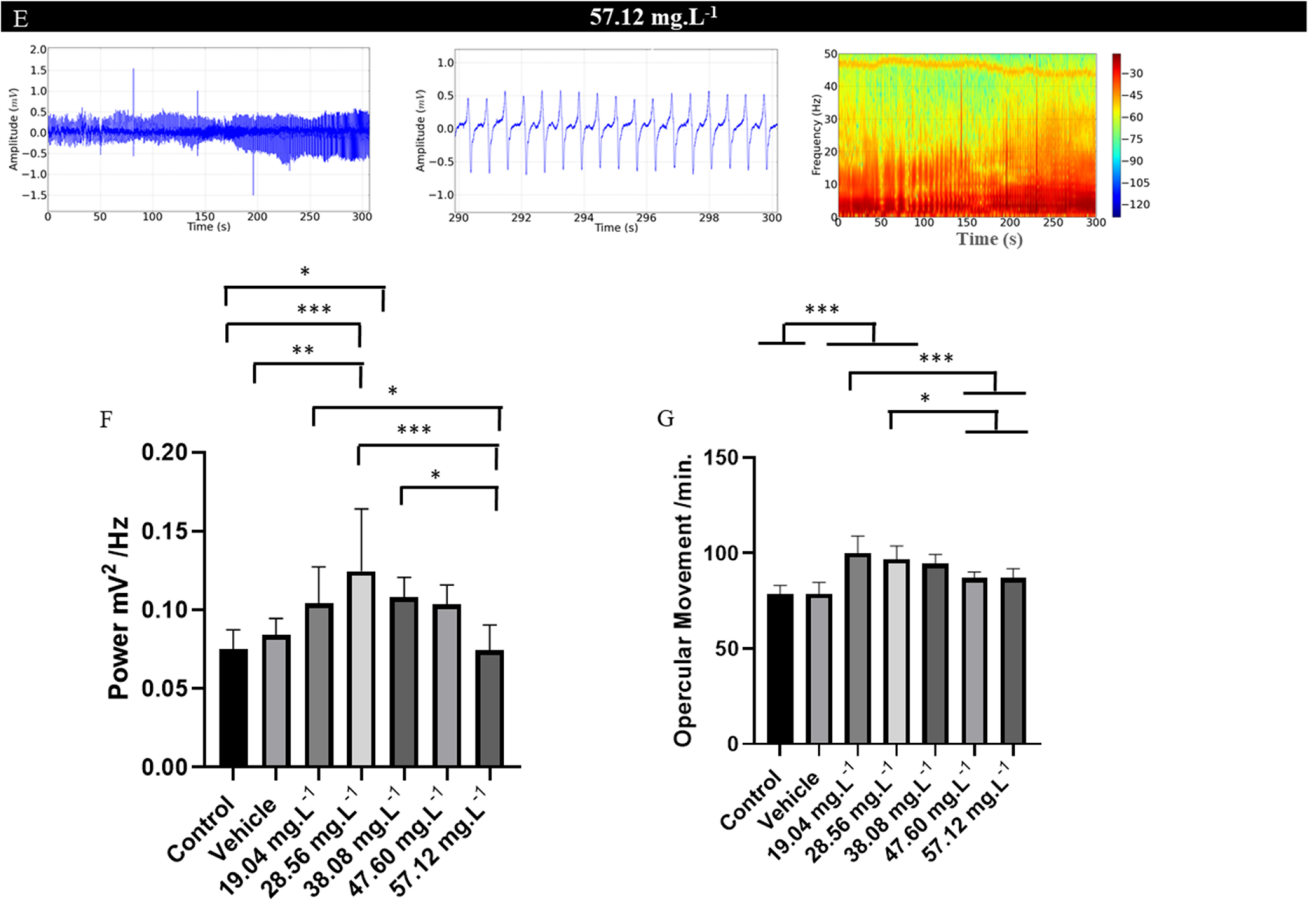
Recording of opercular movement during the recovery period (left), amplification of the recording in the last 10 s (290–300 s) (center) and spectrogram demonstrating the power intensity up to 50 Hz during recovery after OVEO treatment (lasting 5 min) (left), After the following treatments: 19.04 mg. L^−1^ (A), 28.56 mg. L^−1^ (B), 38.08 mg. L^−1^ (C), 47.60 mg. L^−1^ (D) and 57.12 mg. L^−1^ (E). Graph shows the average power values ​​of the opercular movement (mV.^2^/Hz) (F) and average values ​​of opercular movement per minute (G). (ANOVA followed by Tukey's test; *P < 0.05, **p < 0.01, ***p < 0.001; *n* = 9)

During the anesthesia recovery period, the opercular movement frequency demonstrated pharmacological reversibility, with an increase in the frequency of opercular movement and power of the recordings in the spectrograms (Figs. 7A, B, C, D, and E).

The power in the opercular movement recordings for the control group was 0.075 ± 0.012 mV2/Hz, similar to the vehicle group (p = 0.965) and the group treated with 19.04 mg. L^−11^ (= 0.058), 47.60 mg. L^−11^ (= 0.064), and 57.12 mg. L^−11^ (= 0.999), but lower than the 28.56 mg. L^−1^ and 38.08 mg. L^−1^ groups. The group treated with 47.60 mg. L^−1^ (0.103 ± 0.012 mV2/Hz) was similar to the 19.04 mg. L^−1^ (p = 0.999), 28.56 mg. L^−1^ (p = 0.341), 47.60 mg. L^−1^ (p = 0.999), and 57.12 mg. L^−1^ (p = 0.052) groups (Fig. 7F).

The control group had a mean of 78.67 ± 4.35 opercular movements per minute, which was similar to the vehicle (p = 0.999), 47.60 mg. L^−1^ (p = 0.066), and 57.12 mg. L^−1^ (p = 0.066) groups. However, they were lower than the groups treated with 19.04 mg. L-^1^, 28.56 mg. L^−1^, 48.08 mg. L^−1^. The group treated with 38.08 mg. L^−1^ (94.67 ± 7.14 movements per minute) was similar to the group treated with 19.04 mg. L^−1^ (p = 0.385), 28.56 mg. L^−1^ (p = 0.984), 47.60 mg. L^−1^ (p = 0.116) and 57.12 mg. L^−1^ (p = 0.116) (Fig. 7G).

## Discussion

In the Concentration–response context, we observed that above 38.08 µL. L^−1^, we can already observe effects such as a decrease in heart rate and cardiac contractile force, and slower recovery. Gabriel et al. (2022), using post-juvenile Mozambican tilapia, noted that when exposed to concentrations of 100 and 150 µL.L^−^1 of this essential oil from *Origanum vulgare* (Carvacrol 73.15% and Thymol 2.32%), they had deeper anesthesia observed through behavior and opercular beats, in which both were reduced, since Carvacrol potentiates the GABA receptor, by positive allosteric modulation with binding to the α1, β2 and γ2 subunits, a fact similar to the activity of Thymol, which has this sedative effect because it is also a positive allosteric modulator of the GABA receptor, binding to the α1, β3, γ2 and α1, β2 and γ2 subunits (Kessler et al. 2014). However, the discrepancy in the concentrations used in these studies can be justified. due to differences in fish species.

Assuming that species differences can influence the Concentration/response of essential oils and their components, Silva et al. (2019), using juvenile *Colossoma macropomum*, similar to our study, performed anesthetic induction with Lippia origanoides oil (Carvacrol chemotype—~ 47.20%). They observed that at a concentration of 25 µL. L^−1^, there was a longer time to deep anesthesia, as well as loss of the posture reflex. However, recovery was faster and without a significant impact on opercular beats. At a concentration of 47.60 mg. L^−1^, the animals presented a faster induction associated with loss of the posture reflex and a slower recovery compared to the previous concentration. Not only that, there was a significant difference in the reduction of opercular beats, which was further aggravated at concentrations of 100 and 200 µL. L^−1^, data that corroborate our study.

At lower concentrations (19.04, 28.56, and 38.08 mg. L^−1^), anesthetic recovery was faster compared to higher concentrations (47.60 and 57.12 mg. L^−1^), due to two specific factors: the lower concentration required for metabolism in the animal's body and the lipophilic profile of the anesthetic agents in *Origanum vulgare* essential oil, specifically Carvacrol and Tymol, causing faster impregnation effects. Similar results were also found in the study by Silva et al. (2019), which also emphasized the fact that the animals were in the juvenile phase, indirectly influencing metabolic rate, since it can also influence toxicokinetics, in addition to concentration and solubility. This is discussed by Inoue et al. (2011) conducted anesthetic induction in juvenile *C. macropomum* using a eugenol bath. Furthermore, the authors observed that elevated glucose levels (caused by stress) can affect anesthetic distribution and physiological modulation, compromising central nervous system sensitivity. This explains the results found in our study, since the higher the concentration, the higher the glucose elevation observed in our analysis between the control, vehicle, and 38.08 mg. L^−1^ groups.

According to the study by Frota et al. (2022), using thymol and carvacrol supplementation in *Colossoma macropomum* for 30 and 60 days to investigate the effects on performance and plasma changes, they found no significant changes in plasma glucose levels in the fish evaluated. However, the authors observed that fish subjected to higher concentrations of the combination showed a reduction in thrombocytes and lymphocytes, but an increase in eosinophils, suggesting a modulatory effect on some components of the immune system. Furthermore, the same animals demonstrated improved growth performance, indicating that, even without changes in plasma glucose, Thymol and Carvacrol supplementation can positively influence the physiological development of fish. In this context, regarding the glycemic response during anesthesia, the literature does not contain a study specifically investigating *Colossoma macropomum*. However, Bodur et al. (2024) observed similar effects in Nile tilapia, in which low-concentration anesthetic induction has less effect on the glycemic response compared to higher concentrations. This fact is explained by the level of stress during the process of preparing the animal for the procedure (handling, exposure to the anesthetic, possible agitation or hyperventilation), demonstrating that this may be common in more than one species.

Monitoring anesthesia becomes essential for a comprehensive analysis of the condition of fish; the use of electrophysiological data in research allows for a more detailed assessment of anesthetic efficacy (Aydin, 2021).

In this study, it was found that the reduction in cardiac response with a concentration of 19.04 to 38.08 mg. L⁻^1^ presented the best response. Other studies with anesthetics in fish have found similar results, such as that of Vilhena et al. (2022), in which they observed that, with Piper divaricatum essential oil, the 38.08 mg. L⁻^1^ concentration reduced heart rate (HR), but the electrocardiogram components (QRS, Q-T) remained close to control, indicating that cardiac function was still preserved. Similarly, at the 57.12 mg. L^−1^ concentration, similar to this study, 47.60 and 57.12 mg. L⁻^1^ significantly reduced HR, but without hemodynamic repercussions, as sinus rhythm remained.

In this perspective, the study by Vilhena et al. (2022), at 80 µL. L^−1^ the longest latency for total recovery occurred, close to 5 min (300 s), but still with reversibility. Similarly, Reis et al. (2025) using Lippia alba reported that concentrations of 80–100 µL. L⁻^1^ produced moderate bradycardia (32.44% and 33.41%, respectively) without blocks or severe morphological changes, but that 120–140 µL. L⁻^1^ greatly increased the HR reduction (61.25% and 62.71%, respectively) and produced temporary morphological changes, although sinus rhythm returned during recovery. Thus, recovery in the group treated with the highest concentration was slow and concentration-dependent, demonstrating that, at higher concentrations, the substance begins to interfere more profoundly with ventricular electrical activity, exceeding the range considered safe. To corroborate this assertion, a study by Cantanhêde et al. (2021) conducted with *Colossoma macropomum*, subjecting them to short-term exposure to menthol at different dosages (40, 60, and 80 mg/L), found concentrations-dependent bradycardia, with specific prolongation of the RR and QT intervals. The QRS complex showed reduced activity only at the 80 mg/L concentration.

Respiratory rhythm is sensitive to the action of anesthetic agents and can undergo abrupt changes or temporary depression depending on the concentration, duration of exposure, and type of anesthetic used. Therefore, continuous monitoring of the frequency and quality of respiratory activity becomes essential throughout the anesthetic period, allowing for the rapid identification of any physiological impairment (Reis et al. 2024). In a study conducted by Félix et al. (2021), physiological, behavioral, and histological aspects were observed in Nile tilapia anesthetized with MS-222 and propofol. It was found that the samples subjected to MS-22 had an increase in opercular activity, while those subjected to propofol had a reduction in this function (16%). Therefore, it is possible to observe the repercussions of the respiratory center.

In the present study, it was possible to observe that the power of opercular movement at 28.56 mg. L^−1^ was substantially greater compared to other concentrations, although there was no change in respiratory rate. The increased opercular power occurs because, during the anesthetic process, carbon dioxide (CO₂) can accumulate. As a compensatory mechanism, an increase in the power of opercular activity is observed, intensifying ventilation to favor the elimination of excess CO₂ due to stimulation of central and peripheral chemoreceptors. This may reflect a safe option for anesthetic induction in severe respiratory center inhibition, as confirmed by the study by Reis et al. (2024). In an immersion bath with etomidate in juvenile *Colossoma macropomum*, it was shown that the decrease in respiratory rate and power was Concentration-dependent. That is, at lower concentrations (2–3 mg.L^−1^), there was less impact on these functions and a safer recovery. However, at concentrtions of 3.5–4 mg.L^−1^, there was a significant change along with slower recovery, considering a risk to the animal.

In short-term baths with a concentration of 57.12 mg. L^−1^ of *Origanum vulgare* we found that there was no complete return during anesthetic recovery, as a trade-off between the speed of induction vs. speed of recovery, corroborating the study by Gabriel et al. (2022), which in concentrations of 150 µL. L^−1^ in Mozambican tilapia can induce similar effects, given that these factors may differ mainly due to the species or size of the fish, as observed by Can et al. (2019).

Although this study provides the first electrophysiological characterization of OVEO anesthesia in *Colossoma macropomum*, some limitations should be acknowledged. The experimental design was short-term exposures under controlled laboratory conditions, additionally, while electrocardiographic and opercular electromyographic recordings provided valuable insights into cardiac and respiratory modulation, complementary analyses of neural activity or long-term physiological effects were not conducted. Future studies should explore chronic exposure models, neurophysiological mapping, and comparative assessments with other essential oils to better elucidate the mechanisms underlying anesthetic synergy and safety profiles in Amazonian aquaculture species.

Future research should evaluate the potential genotoxic and cytotoxic effects of these anesthetic agents, as well as investigations employing complementary analytical approaches such as DNA damage assays, oxidative stress biomarkers, and molecular or histopathological analyses. This would provide a more comprehensive understanding of the short- and long-term health impacts on fish exposed to acute and chronic conditions.

## Conclusion

Therefore, it is concluded that the use of OVEO as an anesthetic in the fish species *Colossoma macropomun* is safe and effective when used in concentrations of 19.04 to 38.08 mg. L^−1^, providing a therapeutic window in an immersion bath for rapid induction and easy reversibility. For deep anesthesia, concentrations between 47.60 and 57.12 mg. L^−1^ are effective, but they significantly decrease opercular beats, heart rate and contractile strength, and a more prolonged return from anesthesia.

## Data Availability

All data generated or analyzed during this study are included in this published article and its supplementary information files.

## Declarations

### DAS statement request

All data generated or analyzed during this study are included in this published article and its supplementary information files.
